# Sodium acetate attenuates ulcerative colitis by reducing intestinal epithelial apoptosis and remodeling gut microbiota

**DOI:** 10.3389/fmicb.2026.1888741

**Published:** 2026-08-20

**Authors:** Qinxin Liu, Rongshuang Han, Yi Wang, Tao Mao, Xingsi Qi, Bin Cao, Zibin Tian, Yukun Li

**Affiliations:** Department of Gastroenterology, The Affiliated Hospital of Qingdao University, Qingdao, China

**Keywords:** apoptosis, gut microbiota, intestinal barrier, sodium acetate, tight junction proteins, ulcerative colitis

## Abstract

**Background:**

Sodium acetate (NaA), an acetate donor among short-chain fatty acid salts, is involved in intestinal epithelial homeostasis, mucosal inflammation, and host–microbiota interactions. However, its role in ulcerative colitis (UC), particularly in regulating epithelial apoptosis and gut microbiota remodeling, remains unclear. This study investigated the protective effects and potential mechanisms of NaA in experimental colitis.

**Methods:**

LPS-induced intestinal epithelial cell injury models using NCM460 and Caco-2 cells, together with a DSS-induced mouse colitis model, were established to evaluate the effects of NaA *in vitro* and *in vivo*. Cell viability was assessed using the CCK-8 assay. Colonic histopathology and mucus production were examined by H&E and AB/PAS staining. Immunohistochemistry, Western blotting, RT-qPCR, and ELISA were performed to detect barrier-related proteins, inflammatory cytokines, and apoptosis-associated molecules. Gut microbiota alterations were analyzed by 16S rRNA amplicon sequencing.

**Results:**

NaA showed no obvious cytotoxicity within an appropriate concentration range and increased ZO-1 and Occludin expression in intestinal epithelial cells. Under LPS stimulation, NaA restored barrier protein expression and reduced the BAX/BCL-2 ratio and cleaved-caspase-3 expression in both NCM460 and Caco-2 cells. In DSS-induced colitis mice, NaA alleviated body weight loss, disease activity index elevation, colon shortening, and spleen index increase. NaA also attenuated colonic histological injury, increased AB/PAS-positive areas, restored ZO-1, Occludin, and MUC2 expression, and reduced IL-6, TNF-*α*, and IL-1β levels. Moreover, NaA inhibited excessive epithelial apoptosis. 16S rRNA amplicon sequencing and analysis showed that NaA partially reversed DSS-induced gut microbiota dysbiosis, as reflected by improved microbial diversity and community structure, increased Firmicutes/Bacteroidota ratio, reduced *Bacteroides* and *Turicibacter* abundance, and enrichment of potential beneficial genera, including *Bifidobacterium*, *Lactobacillus*, and *Blautia*.

**Conclusion:**

NaA alleviates experimental colitis by suppressing inflammation, inhibiting excessive epithelial apoptosis, preserving epithelial tight-junction and mucus barrier integrity, and partially restoring gut microbiota homeostasis. These findings suggest that acetate-based intervention may be a potential strategy for UC management through coordinated regulation of epithelial protection and microbial remodeling.

## 1 Introduction

Ulcerative colitis (UC) is a chronic, relapsing inflammatory bowel disease primarily affecting the colonic and rectal mucosa, clinically characterized by diarrhea, abdominal pain, mucopurulent bloody stool, and weight loss (Le Berre et al., 2023). The pathogenesis of UC is widely considered multifactorial, involving the interplay of environmental factors, genetic susceptibility, epithelial barrier defects, gut microbiota dysbiosis, and immune dysregulation (Bu F. et al., 2025; Bu S. et al., 2025). Among these, damage to the intestinal mucosal barrier is regarded as a key driver of disease progression. Under physiological conditions, intestinal epithelial cells, intercellular tight junctions, goblet cells and the mucus layer, together with the gut microbiota, maintain mucosal homeostasis (Neurath et al., 2025). The intestinal epithelium renews every 3–5 days, requiring a finely tuned balance between proliferation, differentiation, and apoptosis, In UC, this balance is disrupted: differentiated colonic epithelial cells undergo premature death, whereas immature crypt cells over proliferate, compromising the epithelial barrier and increasing intestinal permeability (Rath and Haller, 2022; Wan et al., 2022; Yang et al., 2026). This facilitates the translocation of luminal microbes and harmful components, which are recognized by pattern recognition receptors on immune cells, triggering the production of cytokines such as TNF-*α* and IFN-*γ*, thereby amplifying inflammation. These inflammatory mediators, in turn, induce additional epithelial apoptosis, creating a vicious cycle that culminates in the clinical manifestations of UC (Abraham et al., 2022; Neurath, 2024). Therefore, strategies aimed at inhibiting excessive epithelial apoptosis and preserving mucosal barrier integrity have emerged as important approaches for ameliorating UC.

Short-chain fatty acids (SCFAs) are saturated fatty acids containing six or fewer carbon atoms, primarily produced through microbial fermentation of resistant starch and dietary fibers in the gut, with acetic acid, propionic acid, and butyric acid accounting for approximately 95% of the total SCFAs (Fusco et al., 2023). SCFAs participate in maintaining epithelial homeostasis, modulating immune responses, and controlling inflammation through multiple mechanisms, including G protein-coupled receptor (GPCR) signaling, histone deacetylase (HDAC) inhibition, and regulation of cellular metabolism (Parada Venegas et al., 2019). In the intestine, SCFAs serve as energy substrates for epithelial cells, promote the expression of tight junction proteins and mucus secretion, thereby enhancing barrier integrity, and influence the differentiation and function of various immune cells such as neutrophils, macrophages, dendritic cells, and regulatory T cells, contributing to mucosal immune homeostasis (Facchin et al., 2024; Mukhopadhya and Louis, 2025). Beyond local intestinal effects, SCFAs also impact multiple organs; for example, they can ameliorate allergic airway inflammation and participate in gut–lung axis regulation (Mann et al., 2024); promote skin barrier-related metabolism and differentiation to reduce early allergen sensitization (Trompette et al., 2022); and exert protective effects on neuroinflammation and the brain’s innate immune system (Wachamo and Gaultier, 2025). Previous studies have reported that SCFA levels are often reduced in inflammatory bowel disease (IBD), accompanied by microbial dysbiosis, mucus barrier impairment, and persistent inflammation (Peng et al., 2022; Xu et al., 2022; Shin et al., 2023). Notably, most research has focused on butyrate, while acetate has received comparatively less attention. Acetate, however, is the most abundant and broadly sourced SCFA in the intestinal lumen (Mukhopadhya and Louis, 2025), serving as an important cellular biofuel and nutrient (Zhang et al., 2023). It contributes to energy and metabolic homeostasis by influencing lipid metabolism and glucose regulation in animals and humans, mitigates oxidative and mitochondrial stress, modulates immune function, and affects tissue and organ physiology, including body weight control and insulin sensitivity (Portincasa et al., 2022; Sankarganesh et al., 2025). Despite its abundance and potential, the mechanisms by which acetate exerts protective effects in UC remain poorly understood, particularly regarding its ability to simultaneously restore mucosal barrier integrity, inhibit excessive epithelial apoptosis, and modulate UC-associated gut microbiota dysbiosis. Sodium acetate (NaA), as a stable form of acetate, offers experimental convenience and serves as a suitable intervention to investigate acetate’s protective effects in UC. Based on this rationale, the present study employed LPS-induced NCM460 and Caco-2 cell injury models *in vitro* and a DSS-induced colitis mouse model *in vivo* to evaluate the protective effects of NaA. By assessing tight junction proteins, inflammatory cytokines, apoptosis-related molecules, and gut microbiota composition, this study aims to elucidate the role of NaA in maintaining intestinal barrier function and provide experimental evidence supporting its potential application in UC prevention and therapy.

## 2 Materials and methods

### 2.1 Reagents and materials

Human normal colonic epithelial cells (NCM460) and human colorectal adenocarcinoma-derived intestinal epithelial cells (Caco-2) were purchased from Procell Life Science & Technology Co., Ltd. (Wuhan, China). NaA was obtained from Shanghai Yuanye Biotechnology Co., Ltd. (Shanghai, China). Lipopolysaccharide (LPS) was purchased from Beyotime Biotechnology Co., Ltd. (Shanghai, China). Dulbecco’s modified Eagle medium (DMEM), fetal bovine serum (FBS), penicillin–streptomycin solution, Phosphate-Buffered Saline (PBS), and trypsin–EDTA were obtained from Procell Life Science & Technology Co., Ltd. (Wuhan, China). The Cell Counting Kit-8 (CCK-8) was purchased from Solarbio Technology Co., Ltd. (Beijing, China). Reagents for RNA extraction, reverse transcription, and reverse transcription-quantitative PCR (RT-qPCR) were purchased from Accurate Biology (Hunan, China). The Bicinchoninic Acid (BCA) Protein Assay Kit was obtained from Elabscience Biotechnology Co., Ltd. (Wuhan, China), and the ECL chemiluminescent detection kit was purchased from Affinity Biosciences (Jiangsu, China). Dextran sulfate sodium (DSS) was obtained from Meilun Bio (Dalian, China). Enzyme-Linked Immunosorbent Assay (ELISA) kits for Interleukin-6 (IL-6), Tumor Necrosis Factor-alpha (TNF-*α*), and Interleukin-1 beta (IL-1β) were purchased from Ruixin Biotech (Quanzhou, China). Primary antibodies against ZO-1 were purchased from Abcam (Cambridge, UK), Occludin from Proteintech (Wuhan, China), and MUC2, BAX, BCL-2, and cleaved-caspase-3 from Cell Signaling Technology (Danvers, MA, USA). GAPDH antibody was obtained from Huabio (Hangzhou, China). Detailed information on the reagents, kits, and antibodies used in this study, including suppliers, catalog numbers, and antibody dilution ratios, is provided in Supplementary Table S1.

### 2.2 Cell culture and treatment

NCM460 cells and Caco-2 cells were cultured in DMEM supplemented with 10% FBS and 1% penicillin–streptomycin solution in a humidified incubator at 37 °C with 5% CO₂. Cells were used for subsequent experiments when confluence reached 70–80%. Cell viability in response to NaA and LPS was first evaluated in NCM460 cells using the CCK-8 assay. Cells were seeded into 96-well plates and allowed to adhere, then treated with varying concentrations of NaA (0, 5, 10, 20, 40, 80 mM) or LPS (0, 1, 10, 30, 50, 100 μg/mL) for 12 or 24 h. After treatment, CCK-8 working solution was added according to the manufacturer’s instructions and incubated for 1 h. Absorbance was measured at 450 nm to assess cell viability. For NaA concentration screening and LPS-induced injury condition determination, NCM460 cells were initially used. Cells were treated with different concentrations of NaA for 24 h, followed by collection of total protein to evaluate the expression of ZO-1 and Occludin, in order to determine the optimal intervention concentration. For LPS concentration screening, cells were serum-starved overnight and then treated with varying concentrations of LPS for 12 h. Protein was collected to assess ZO-1 and Occludin expression, identifying the appropriate injury-inducing concentration. Based on preliminary experiments, 20 mM NaA and 100 μg/mL LPS were selected for subsequent experiments in both NCM460 and Caco-2 cells. In formal experiments, cells were divided into three groups: Control, LPS, and LPS + NaA. The Control group received an equal volume of culture medium; the LPS group was treated with 100 μg/mL LPS for 12 h; the LPS + NaA group was pretreated with 20 mM NaA for 12 h, followed by 100 μg/mL LPS for an additional 12 h. After treatments, cells were collected for total protein or RNA extraction, which were used for Western blot and RT-qPCR analyses, respectively. All cell experiments were independently repeated at least three times.

### 2.3 Animal experiment

Male C57BL/6 mice (6 weeks old) were housed under specific pathogen-free (SPF) conditions with ad libitum access to food and water, under a 12 h light/dark cycle. All animal procedures were approved by the Animal Ethics Committee of the Affiliated Hospital of Qingdao University (Ethics No.: AHQU-MAL20251105LQX). After 1 week of acclimatization, mice were randomly assigned to four groups (*n* = 6 per group): Control, DSS model, low-dose sodium acetate (L-NaA), and high-dose sodium acetate (H-NaA). The Control group received plain water throughout the experiment. The DSS group was untreated for the first 7 days and then given 2.5% DSS solution ad libitum for the following 7 days. The L-NaA group was administered 0.2 mL of 150 mM NaA daily by oral gavage for 7 days, followed by continued NaA gavage combined with ad libitum 2.5% DSS solution for the next 7 days. The H-NaA group received 0.2 mL of 300 mM NaA daily, with the same DSS treatment as the L-NaA group. During the experiment, body weight, stool consistency, and fecal blood were recorded daily. On day 15, after overnight fasting, mice were anesthetized with 3% isoflurane and then euthanized by cervical dislocation. Death was confirmed by the cessation of respiration and heartbeat before tissue collection. The entire colon was excised and its length measured. Cecal contents were aseptically collected into sterile cryotubes, snap-frozen in liquid nitrogen, and stored at −80 °C for subsequent gut microbiota analysis. Portions of colon tissue were fixed in 4% paraformaldehyde for hematoxylin and eosin (H&E), Alcian blue/periodic acid–Schiff (AB/PAS), and immunohistochemical analyses, while the remaining tissue was snap-frozen in liquid nitrogen and stored at −80 °C for Western blot, RT-qPCR, and ELISA analyses.

### 2.4 Disease activity index (DAI) assessment

During the experimental period, body weight, stool consistency, and fecal blood of mice were monitored daily to calculate the disease activity index (DAI). The scoring criteria are summarized in Table 1 (Kihara et al., 2003). DAI = (weight loss score + stool consistency score + bleeding score)/3.

**Table 1:** Disease activity index (DAI) scoring system.

Score	Weight loss	Stool consistency	Rectal bleeding
0	None	Normal	Normal
1	1–5%	Soft stool	Occult blood
2	6–10%	Mucus-like stool	Slight bleeding
3	11–15%	Thin liquid stool	Blood in the stool
4	>15%	Watery diarrhea	Gross bleeding in the stool

### 2.5 Hematoxylin and eosin (H&E) and Alcian blue/periodic acid–Schiff (AB/PAS)

Colon tissues from each group were fixed in 4% paraformaldehyde for 24–48 h, followed by routine paraffin embedding and sectioning at 4 μm thickness. For H&E staining, sections were deparaffinized in xylene, rehydrated through a graded ethanol series, and then sequentially stained with hematoxylin, differentiated, blued, and counterstained with eosin. After dehydration, clearing, and mounting, histopathological changes in the colon were examined under a light microscope. The detailed histological damage scoring criteria for H&E-stained colon sections are provided in Supplementary Table S2. For AB/PAS staining, sections were deparaffinized and rehydrated, then processed according to the manufacturer’s instructions, including Alcian blue staining, periodic acid treatment, and Schiff reagent development, to evaluate goblet cell density and mucus secretion in the colonic mucosa. For H&E and AB/PAS staining analyses, five microscopic fields were randomly selected from each section for evaluation, and the averaged value from these fields was used as the individual data point for each mouse. All images were captured under identical conditions, and histopathological and goblet cell evaluations were performed by an investigator blinded to the experimental groups.

### 2.6 Immunohistochemistry (IHC)

Paraffin-embedded colon sections were baked at 60 °C for 2 h, deparaffinized in xylene, and rehydrated through a graded ethanol series. Antigen retrieval was performed using either citrate buffer or Tris-EDTA buffer. Endogenous peroxidase activity was blocked with 3% hydrogen peroxide for 10–15 min, followed by PBS washing and blocking with 5% goat serum at room temperature for 30–60 min. Sections were then incubated overnight at 4 °C in a humidified chamber with primary antibodies against ZO-1, Occludin, or MUC2. After washing with PBS the next day, sections were incubated with horseradish peroxidase (HRP)-conjugated secondary antibodies at room temperature for 30–60 min. Immunoreactivity was visualized using 3,3′-diaminobenzidine (DAB), followed by hematoxylin counterstaining, dehydration, clearing, and mounting with neutral resin. Sections were examined under a light microscope, and images were captured. Five random fields per section were analyzed using ImageJ software to quantify the positive staining area.

### 2.7 Western blot analysis

Appropriate amounts of colon tissue or cell samples were lysed on ice using radioimmunoprecipitation assay (RIPA) buffer containing protease inhibitors. Lysates were centrifuged at 12,000 rpm for 15 min at 4 °C, and the supernatant was collected. Protein concentration was determined using the BCA assay. Equal amounts of protein were mixed with 5 × loading buffer, denatured at 95 °C for 5–10 min, and separated by 10% sodium dodecyl sulfate-polyacrylamide gel electrophoresis (SDS-PAGE), followed by transfer onto PVDF membranes. Membranes were blocked with 5% non-fat milk at room temperature for 1 h and then incubated overnight at 4 °C with primary antibodies against ZO-1, Occludin, BAX, BCL-2, cleaved-caspase-3, or GAPDH. The following day, membranes were incubated with appropriate HRP-conjugated secondary antibodies at room temperature for 1 h. Protein bands were visualized using ECL chemiluminescent detection and captured with a gel imaging system. Band intensities were quantified using ImageJ software and normalized to GAPDH as the internal control.

### 2.8 Reverse transcription-quantitative PCR (RT-qPCR)

Total RNA was extracted from colon tissues or cells using TRIzol reagent according to the manufacturer’s instructions. RNA concentration and purity were measured using a micro-volume spectrophotometer. Equal amounts of total RNA were reverse-transcribed into complementary DNA (cDNA). RT-qPCR was performed using SYBR Green premix on a real-time PCR system. The PCR reaction volume was 20 μL, containing cDNA template, forward and reverse primers, SYBR Green premix, and nuclease-free water. The amplification program was as follows: initial denaturation at 95 °C for 5 min, followed by 40 cycles of 95 °C for 10 s and 60 °C for 30 s. Each sample was run in triplicate. *GAPDH* was used as the internal reference gene, and relative expression levels of target genes were calculated using the 2^-ΔΔCt^ method. Primer sequences for target genes in cells and mice are listed in Tables 2, 3, respectively.

**Table 2:** Primer sequences used for RT-qPCR in NCM460 cells.

Gene	Forward primer (5′ → 3′)	Reverse primer (5′ → 3′)
*ZO-1*	GACCAATAGCTGATGTTGCCAGAG	TGCAGGCGAATAATGCCAGA
*Occludin*	AGCAGCGGTGGTAACTTTGAG	CCTCCAGCTCATCACAGGACTC
*BAX*	TGCTTCAGGGTTTCATCCA	GACACTCGCTCAGCTTCTTG
*BCL-2*	ATACCCTAATCCTCAACCC	GCTCAAATACTGCCTCTGT
*GAPDH*	GCACCGTCAAGGCTGAGAAC	TGGTGAAGACGCCAGTGGA

**Table 3:** Primer sequences used for RT-qPCR in mouse colon tissues.

Gene	Forward primer (5′ → 3′)	Reverse primer (5′ → 3′)
*ZO-1*	CCACCTCTGTCCAGCTCTTC	CACCGGAGTGATGGTTTTCT
*Occludin*	AGCCTCGGTACAGCAGCAAT	CCCACCTGTCGTGTAGTCTGT
*IL-6*	AGACGCATCTCAGCTGGTAAA	TTTGGGGGAGGATGTTTGGAT
*IL-1β*	GAAATGCCACCTTTTGACAGTG	TGGATGCTCTCATCAGGACAG
*TNF-α*	AAGTGTTCCCACACCTCTCTC	TGCACTTAGACCCCTTTCCTC
*GAPDH*	GTATGACTCCACTCACGGCAAA	GGTCTCGCTCCTGGAAGATG

### 2.9 ELISA detection of inflammatory cytokines

Appropriate amounts of colon tissue were homogenized on ice in pre-cooled PBS and centrifuged at 12,000 rpm for 15 min at 4 °C. The supernatant was collected, and total protein concentration was determined using the BCA assay for normalization. Levels of IL-6, TNF-*α*, and IL-1β were measured using corresponding ELISA kits according to the manufacturer’s instructions. Standards and samples were added to the designated wells of the microplate, followed by sequential incubation, washing, addition of enzyme conjugates and substrate, and termination of the reaction. Absorbance was measured at 450 nm, and cytokine concentrations were calculated based on the standard curve and normalized to total protein concentration.

### 2.10 16S rRNA amplicon sequencing and analysis

At the end of the experiment, cecal content samples from each group of mice were aseptically collected into sterile cryotubes, snap-frozen in liquid nitrogen, and stored at −80 °C. Total bacterial genomic DNA was extracted from cecal content samples using a microbial DNA extraction kit. DNA concentration and purity were assessed using a NanoDrop micro-spectrophotometer, and DNA integrity was evaluated by agarose gel electrophoresis. The V3–V4 hypervariable regions of the bacterial 16S rRNA gene were amplified using the extracted DNA as a template with the primers 338F (5′-ACTCCTACGGGAGGCAGCA-3′) and 806R (5′-GGACTACHVGGGTWTCTAAT-3′). PCR products were purified, quantified, and pooled in equimolar amounts to construct sequencing libraries, which were subjected to paired-end sequencing on the Illumina platform. Sequencing was performed by Majorbio Bio-Pharm Technology Co., Ltd. (Shanghai, China). Raw sequences were quality-filtered, merged, and subjected to chimera removal using the Majorbio standard analysis pipeline to generate amplicon sequence variants (ASVs). Representative ASV sequences were taxonomically annotated based on the SILVA database. Alpha diversity was assessed using the ACE, Chao1, Shannon, and Simpson indices, while beta diversity was analyzed via principal coordinate analysis (PCoA) and non-metric multidimensional scaling (NMDS) based on ASV-level data. Differential taxa were identified using linear discriminant analysis effect size (LEfSe) analysis, and *p* < 0.05 was considered statistically significant.

### 2.11 Statistical analysis

All data were analyzed using GraphPad Prism 10.6.0. Quantitative data are presented as mean ± standard deviation (mean ± SD). Comparisons between two groups were performed using an unpaired Student’s *t*-test, while comparisons among multiple groups were conducted using one-way analysis of variance (ANOVA) followed by Tukey’s *post hoc* test for pairwise comparisons. *p* < 0.05 was considered statistically significant.

## 3 Results

### 3.1 Screening of optimal NaA concentration and determination of LPS-induced injury conditions

**Figure 1:**
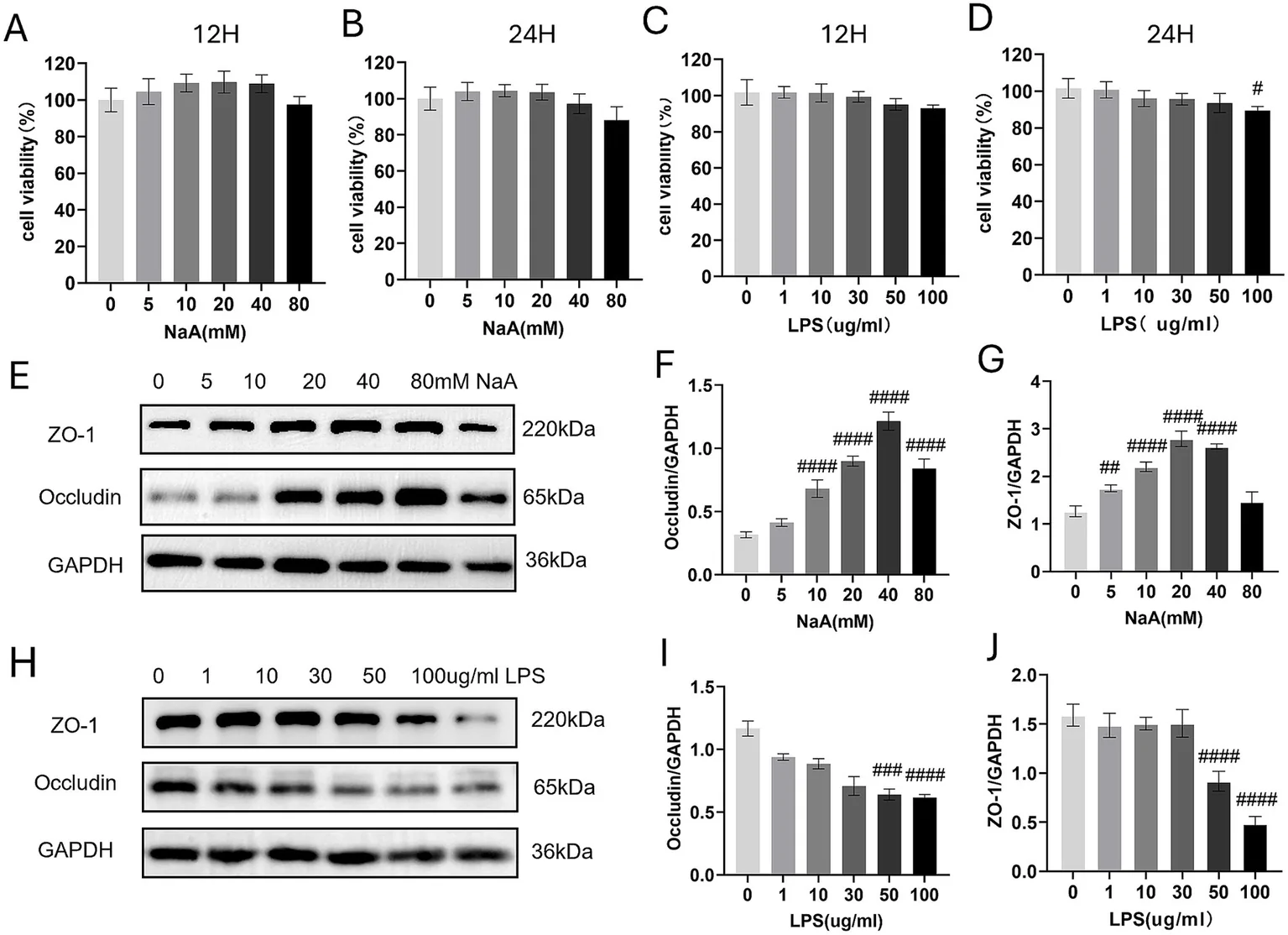
Screening of optimal NaA concentration and determination of LPS-induced injury conditions. **(A,B)** Cell viability of NCM460 cells after treatment with different concentrations of NaA (0, 5, 10, 20, 40, 80 mM) for 12 and 24 h. **(C,D)** Cell viability of NCM460 cells after treatment with different concentrations of LPS (0, 1, 10, 30, 50, 100 μg/mL) for 12 and 24 h. **(E–G)** Relative expression of ZO-1 and Occludin in NCM460 cells treated with varying NaA concentrations, determined by Western blotting. **(H–J)** Relative expression of ZO-1 and Occludin in NCM460 cells treated with varying LPS concentrations, determined by Western blotting. Data are presented as mean ± SD (*n* = 3). #*p* < 0.05, ##*p* < 0.01, ###*p* < 0.001, ####*p* < 0.0001 compared to 0 mM NaA or 0 μg/mL LPS.

To determine the optimal concentration of NaA for intestinal epithelial cells, the effects of varying NaA concentrations on NCM460 cell viability were first assessed using the CCK-8 assay. The results showed that treatment with 5–40 mM NaA for 12 or 24 h did not induce significant cytotoxicity, whereas exposure to 80 mM NaA for 24 h led to a slight reduction in cell viability, which nevertheless remained at a relatively high level (Figures 1A,B). Subsequently, the impact of different LPS concentrations on NCM460 cell viability was evaluated. Treatment with 1–50 μg/mL LPS for 12 or 24 h caused minimal effects on cell viability, while 100 μg/mL LPS for 24 h significantly decreased viability (Figures 1C,D). The effects of NaA and LPS on tight junction protein expression were further examined. Western blot analysis indicated that increasing concentrations of NaA gradually upregulated the expression of ZO-1 and Occludin, with maximal promotion observed at 20–40 mM, and a slight decline at 80 mM (Figures 1E–G). In contrast, LPS treatment led to a dose-dependent reduction in ZO-1 and Occludin levels, with more pronounced decreases at 50–100 μg/mL (Figures 1H–J). These results suggest that NaA within a certain concentration range can enhance the expression of epithelial barrier proteins, whereas higher LPS concentrations reliably induce epithelial barrier injury. Based on the combined evaluation of cell viability and protein expression, 20 mM NaA and 100 μg/mL LPS were selected for subsequent *in vitro* experiments.

### 3.2 NaA reverses LPS-induced barrier damage and attenuates apoptosis in intestinal epithelial cells

**Figure 2:**
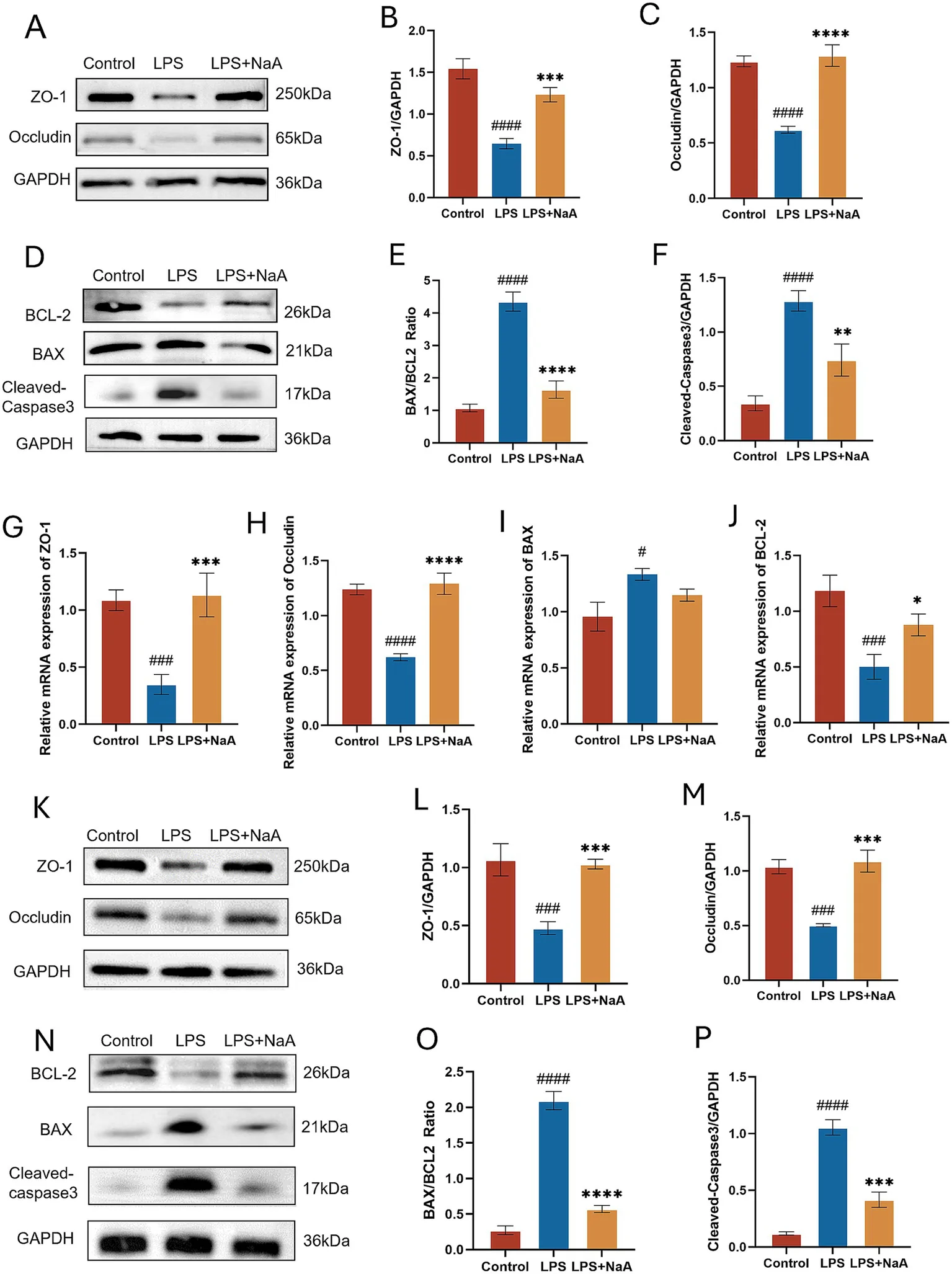
NaA attenuates LPS-induced barrier damage and apoptosis in intestinal epithelial cells. **(A–C)** Representative Western blot images of ZO-1 and Occludin and corresponding densitometric analyses in NCM460 cells. **(D–F)** Representative Western blot images of BCL-2, BAX, and cleaved-caspase-3 and corresponding densitometric analyses in NCM460 cells. **(G–J)** RT-qPCR analysis of *ZO-1*, *Occludin, BAX* and *BCL-2* mRNA expression in NCM460 cells. **(K–M)** Representative Western blot images of ZO-1 and Occludin and corresponding densitometric analyses in Caco-2 cells. **(N–P)** Representative Western blot images of BCL-2, BAX, and cleaved-caspase-3 and corresponding densitometric analyses in Caco-2 cells. Data are presented as mean ± SD (*n* = 3). #*p* < 0.05, ##*p* < 0.01, ###*p* < 0.001, ####*p* < 0.0001 compared to Control group; *p < 0.05, **p < 0.01, ***p < 0.001, ****p < 0.0001 compared to LPS group.

After determining the appropriate concentrations of NaA and LPS using NCM460 cells, the protective effects of NaA were evaluated in both NCM460 and Caco-2 intestinal epithelial cell models under the same experimental conditions. In NCM460 cells, LPS treatment significantly decreased the protein expression of ZO-1 and Occludin compared with the control group, whereas NaA intervention markedly restored their expression levels (Figures 2A–C). Meanwhile, LPS exposure significantly increased the BAX/BCL-2 ratio and cleaved-caspase-3 expression, while NaA treatment alleviated these apoptotic alterations (Figures 2D–F). Consistently, RT-qPCR analysis showed that LPS significantly downregulated *ZO-1* and *Occludin* mRNA expression and increased *BAX* mRNA expression while decreasing *BCL-2* mRNA expression in NCM460 cells. These alterations were partially reversed after NaA treatment (Figures 2G–J). Consistent protective effects were also observed in Caco-2 cells. LPS stimulation reduced ZO-1 and Occludin protein expression, whereas NaA treatment restored the levels of these barrier-related proteins (Figures 2K–M). In addition, NaA significantly decreased the LPS-induced elevation of the BAX/BCL-2 ratio and cleaved-caspase-3 expression in Caco-2 cells (Figures 2N–P). Collectively, these results demonstrate that NaA protects intestinal epithelial cells from LPS-induced barrier disruption and excessive apoptosis.

### 3.3 NaA alleviates overall disease phenotypes in DSS-induced colitis mice

**Figure 3:**
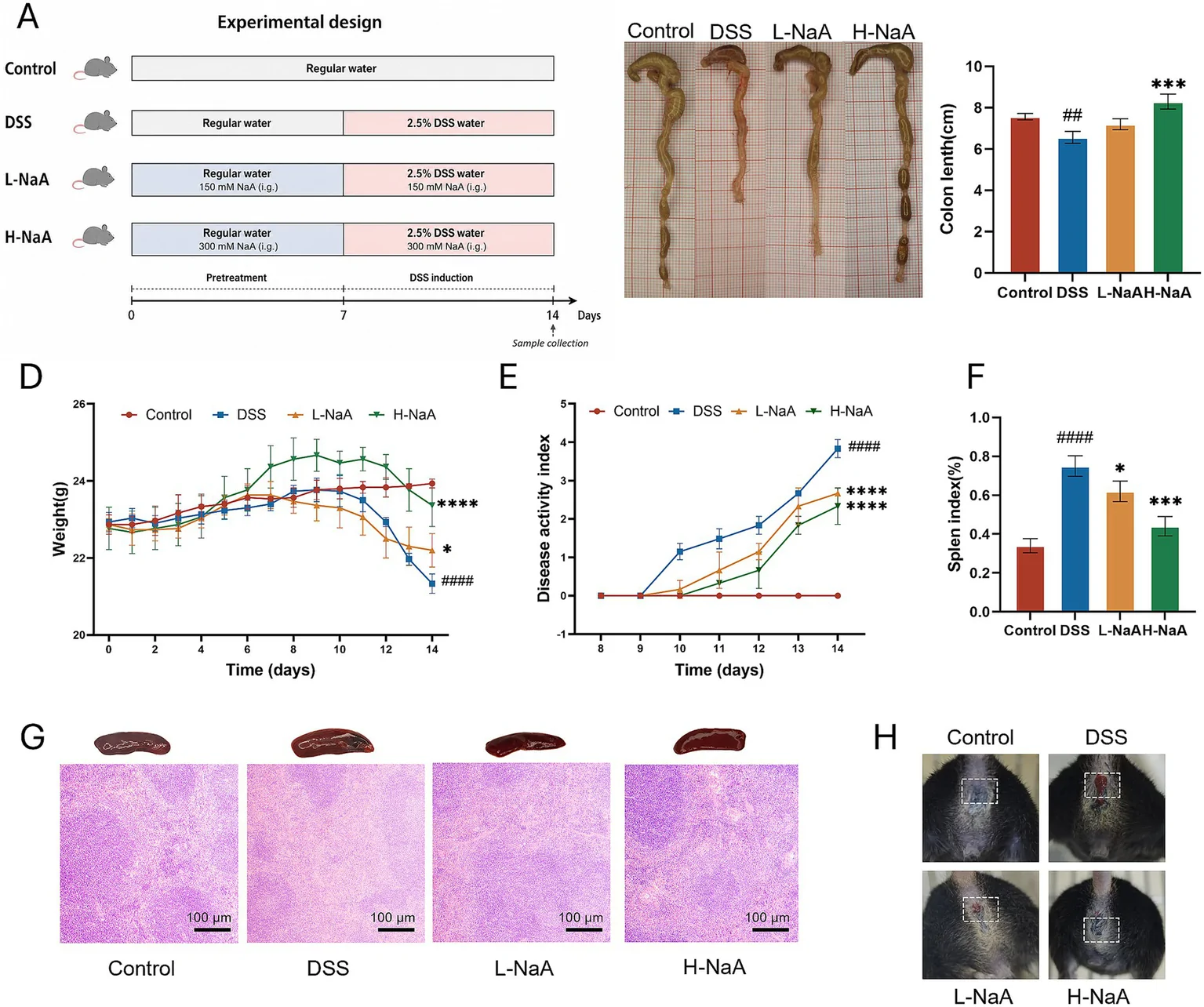
NaA ameliorates gross disease manifestations in DSS-induced colitis mice. **(A)** Schematic diagram of the experimental design. **(B)** Representative images of mouse colons. **(C)** Quantitative analysis of colon length. **(D)** Body weight changes of mice during the experiment. **(E)** Disease activity index (DAI) scores. **(F)** Comparison of spleen index among groups. **(G)** Representative images of spleens and H&E-stained sections. **(H)** Severity of fecal blood. Data are presented as mean ± SD (*n* = 6). ##*p* < 0.01, ####*p* < 0.0001 compared to Control group; *p < 0.05, ***p < 0.001, ****p < 0.0001 compared to DSS group.

To evaluate the protective effects of NaA in experimental colitis, a DSS-induced mouse model of UC was established, and mice were administered low- or high-dose NaA (Figure 3A). Compared with the control group, mice in the DSS group exhibited significant colon shortening, whereas NaA intervention increased colon length, with more pronounced improvement observed in the high-dose group (Figures 3B,C). Analysis of body weight showed that DSS treatment induced a marked reduction in body weight during the later stages, whereas NaA intervention mitigated weight loss, with the high-dose group demonstrating greater recovery (Figure 3D). DSS administration also significantly increased the disease activity index (DAI) scores, which were markedly reduced after NaA treatment (Figure 3E). In addition, DSS treatment significantly increased the spleen index, indicating systemic inflammatory activation, whereas NaA treatment reduced the spleen index compared with the DSS group (Figure 3F). Consistently, representative spleen images and H&E-stained sections showed that DSS treatment induced splenic enlargement accompanied by inflammatory alterations, including increased cellular accumulation and disrupted splenic architecture, whereas NaA intervention partially alleviated these pathological changes and restored the overall splenic morphology (Figure 3G). Furthermore, gross inspection showed that fecal blood was evident in DSS-treated mice, while NaA treatment alleviated this symptom (Figure 3H). Collectively, these results indicate that NaA markedly improves general clinical manifestations in DSS-induced colitis mice.

### 3.4 NaA attenuates colonic tissue damage and partially preserves goblet cells and mucus layer

**Figure 4:**
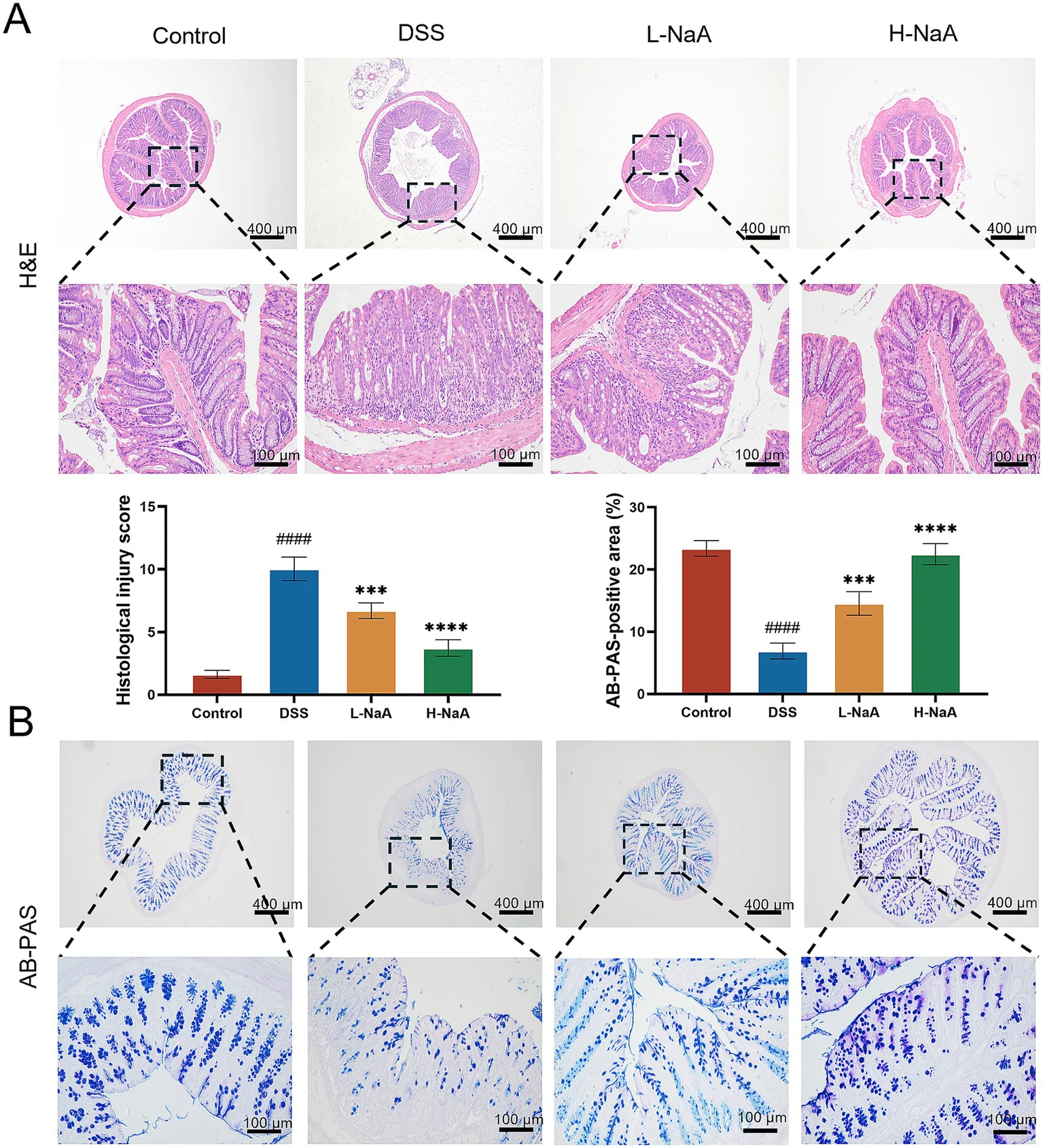
NaA attenuates DSS-induced colonic tissue damage and restores goblet cells. **(A)** Representative H&E-stained colon sections and corresponding histological injury scores for each group. **(B)** Representative AB/PAS-stained colon sections and quantification of positive areas. Data are presented as mean ± SD (*n* = 6). ####*p* < 0.0001 compared to control group; ***p < 0.001, ****p < 0.0001 compared to DSS group.

H&E staining showed that colonic mucosa in the control group was intact, with well-organized crypts and minimal inflammatory cell infiltration. In contrast, DSS-treated mice exhibited pronounced mucosal damage, disrupted crypt architecture, and extensive inflammatory cell infiltration. NaA intervention mitigated these pathological changes, with more pronounced restoration of colonic morphology observed in the high-dose group (Figure 4A). Histological scoring further confirmed these findings, showing a significant increase in tissue injury scores in the DSS group, which were markedly reduced by both low- and high-dose NaA, with the high-dose group showing the greatest improvement (Figure 4A). AB/PAS staining revealed abundant goblet cells and prominent acidic mucin in the control group. DSS treatment significantly reduced AB/PAS-positive areas, indicating goblet cell depletion and impaired mucus secretion. NaA administration significantly increased AB/PAS-positive areas, with the high-dose group exhibiting the largest recovery (Figure 4B). Collectively, these results indicate that NaA not only attenuates DSS-induced histological damage in the colon but also partially preserves goblet cells and the mucus layer, suggesting a protective effect on colonic mucosal barrier integrity.

### 3.5 NaA partially restores DSS-induced reduction of colonic barrier protein expression

**Figure 5:**
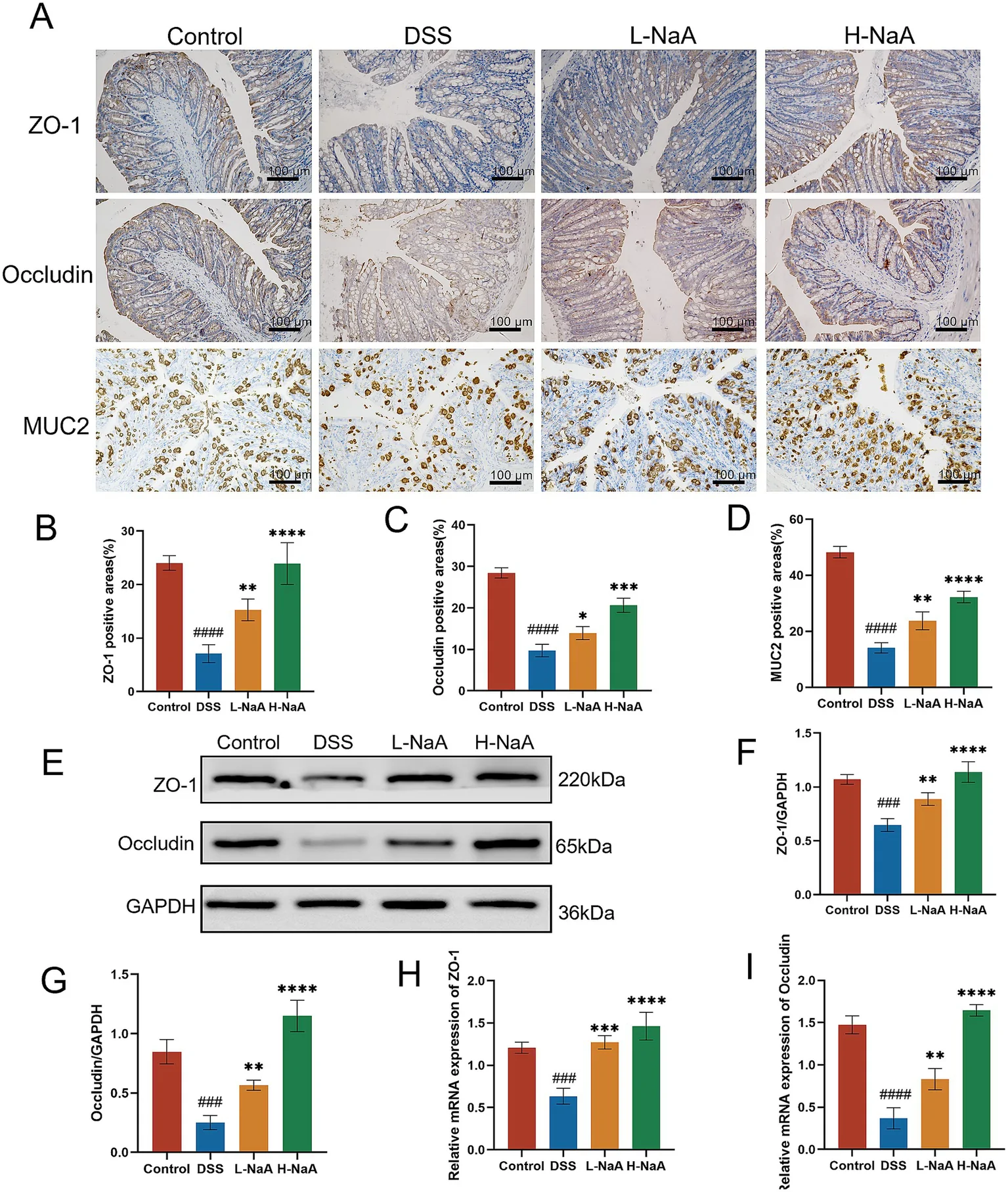
NaA restores the expression of colonic barrier-related proteins in DSS-induced mice. **(A–D)** Representative immunohistochemical images and quantification of ZO-1, Occludin, and MUC2 in colon tissues. **(E–G)** Western blot analysis of ZO-1 and Occludin protein expression and densitometric quantification. **(H,I)** RT-qPCR analysis of *ZO-1* and *Occludin* mRNA expression in colon tissues. Data are presented as mean ± SD (*n* = 3). ###*p* < 0.001, ####*p* < 0.0001 compared to Control group; *p < 0.05, **p < 0.01, ***p < 0.001, ****p < 0.0001 compared to DSS group.

To further evaluate the protective effects of NaA on the intestinal barrier, the expression of ZO-1, Occludin, and MUC2 in colon tissues was examined. Immunohistochemistry results showed that, compared with the control group, DSS treatment markedly reduced the positive staining areas of ZO-1, Occludin, and MUC2. NaA intervention significantly increased their expression, with more pronounced restoration observed in the high-dose group (Figures 5A–D). Western blot analysis further confirmed that DSS significantly downregulated ZO-1 and Occludin protein levels, whereas NaA treatment markedly restored their expression (Figures 5E–G). Consistently, RT-qPCR results demonstrated that DSS significantly decreased *ZO-1* and *Occludin* mRNA levels, which were notably recovered after NaA treatment, with the high-dose group showing the greatest upregulation (Figures 5H,I). Collectively, these results indicate that NaA effectively restores the expression of colonic epithelial barrier proteins impaired by DSS, suggesting a protective role of NaA on intestinal barrier integrity.

### 3.6 NaA attenuates DSS-induced local inflammation and modulates apoptosis-related molecules

**Figure 6:**
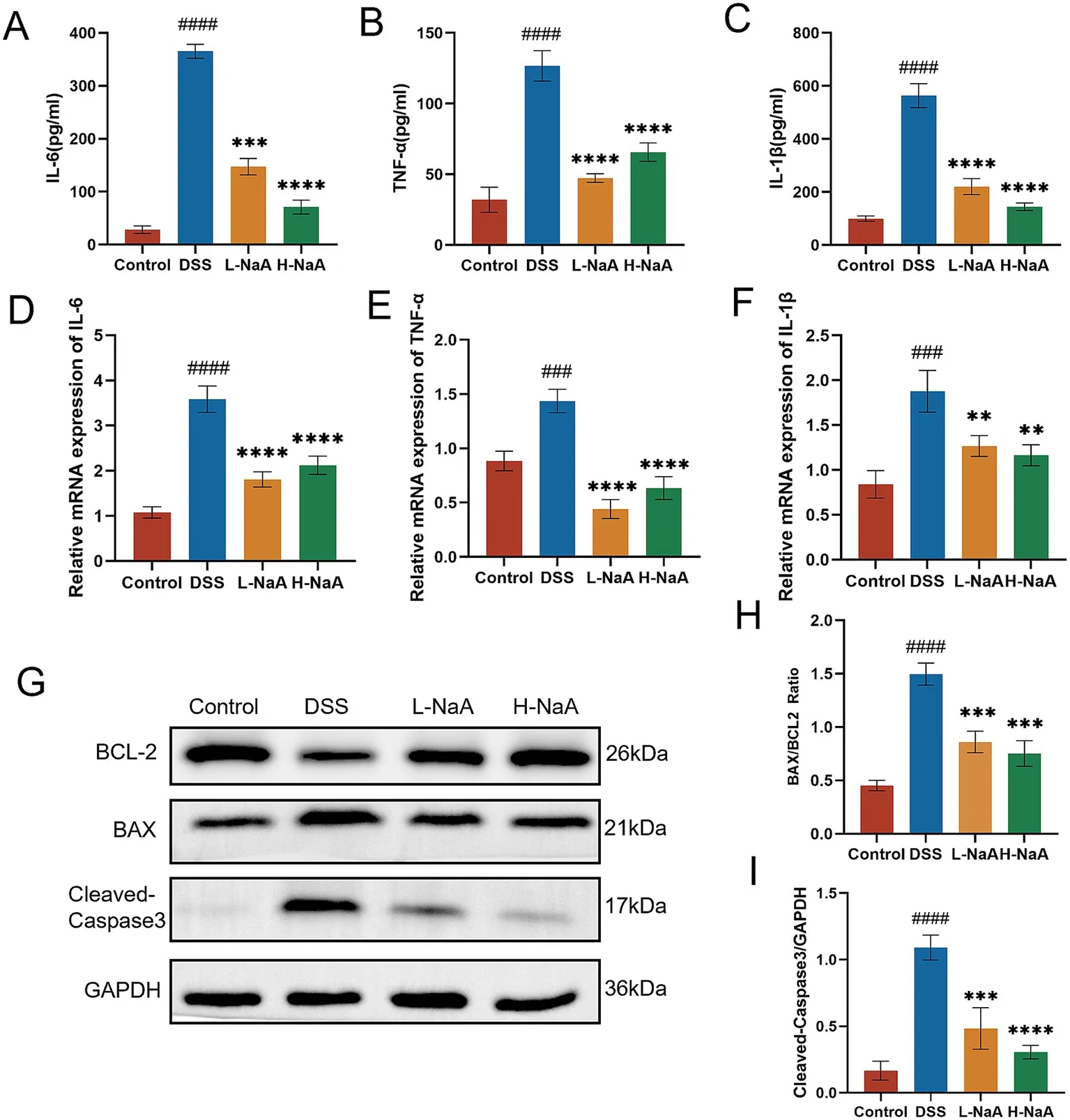
NaA inhibits DSS-induced inflammatory responses and attenuates colonic epithelial apoptosis. **(A–C)** Levels of IL-6, TNF-*α*, and IL-1β proteins in mouse colon tissues. **(D–F)** mRNA expression levels of *IL-6, TNF-α, and IL-1β* in mouse colon tissues. **(G–I)** Protein expression and densitometric analysis of BCL-2, BAX, and cleaved-caspase-3 in colon tissues. Data are presented as mean ± SD (*n* = 3). ###*p* < 0.001, ####*p* < 0.0001 compared to control group; **p < 0.01, ***p < 0.001, ****p < 0.0001 compared to the DSS group.

To evaluate the effects of NaA on colonic inflammation, the expression of IL-6, TNF-*α*, and IL-1β in colon tissues was examined. Protein levels of these inflammatory cytokines were significantly elevated in the DSS group compared with the control group, whereas both low- and high-dose NaA markedly reduced their expression (Figures 6A–C). Consistently, RT-qPCR analysis showed that DSS significantly upregulated *IL-6, TNF-α,* and *IL-1β* mRNA levels, which were notably decreased following NaA intervention (Figures 6D–F). These results indicate that NaA effectively suppresses DSS-induced colonic inflammation. The expression of apoptosis-related proteins was further assessed. Western blot analysis revealed that DSS treatment significantly increased the BAX/BCL-2 ratio and cleaved-caspase-3 protein levels, while NaA intervention markedly reduced these indices (Figures 6G–I), with high-dose NaA exhibiting a more pronounced inhibitory effect on cleaved-caspase-3. Collectively, these findings suggest that NaA not only mitigates the inflammatory response but also inhibits excessive apoptosis of colonic epithelial cells induced by DSS.

### 3.7 NaA partially restores DSS-induced reduction in gut microbial diversity and reshapes the overall microbial structure

**Figure 7:**
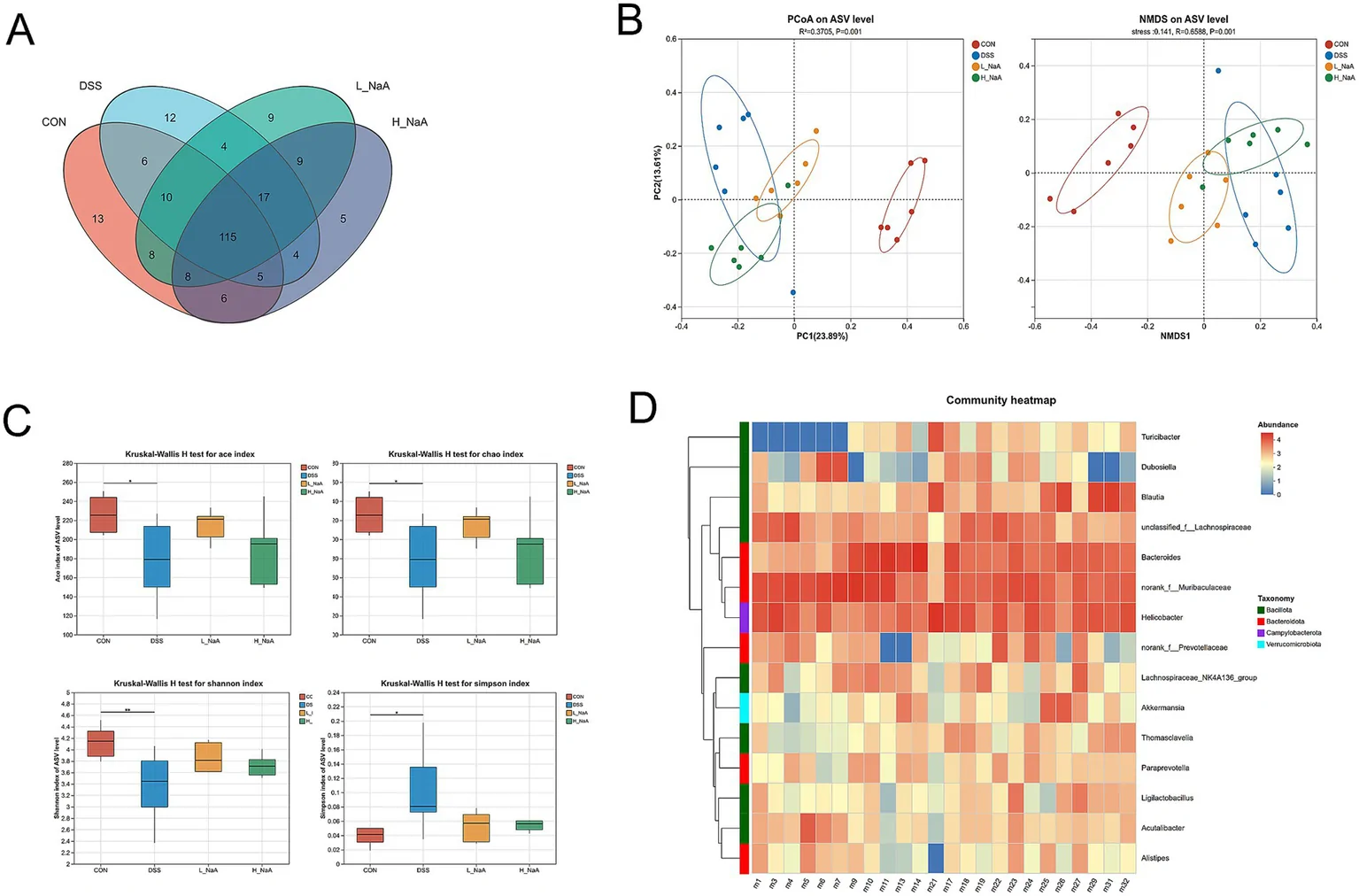
NaA improves DSS-induced gut microbial diversity and community structure. **(A)** Venn diagram showing shared and unique ASVs among groups. **(B)** Principal coordinate analysis (PCoA) and non-metric multidimensional scaling (NMDS) based on ASV-level data. **(C)** Alpha diversity analysis of each group, including ACE, Chao1, Shannon, and Simpson indices. **(D)** Heatmap of dominant bacterial taxa composition in each group. Data are presented as mean ± SD (*n* = 6). *p < 0.05, **p < 0.01, compared to DSS group.

To investigate the regulatory effects of NaA on DSS-induced gut microbiota dysbiosis, cecal content samples from mice in each group were subjected to 16S rRNA amplicon sequencing and analysis. The Venn diagram showed both shared and group-specific ASVs among the different groups, suggesting that both DSS treatment and NaA intervention affected the composition of the gut microbiota (Figure 7A). Beta diversity analysis based on ASV-level data showed that samples from different groups could be clearly distinguished by both PCoA and NMDS analyses. The DSS group was distinctly separated from the control group, whereas NaA treatment shifted the microbial community structure toward that of the control group (Figure 7B). Alpha diversity analysis showed that the ACE, Chao1, and Shannon indices were decreased, whereas the Simpson index was increased in the DSS group compared with the control group, indicating that DSS reduced microbial richness and diversity. After NaA intervention, these alterations showed an alleviating trend to varying degrees, especially in the low-dose NaA group, while the statistical significance varied among different indices (Figure 7C). The community heatmap further revealed obvious differences in the composition of dominant bacterial taxa among the groups (Figure 7D). These results indicate that NaA modulates DSS-induced gut microbiota dysbiosis at the overall community level, with an alleviating trend in alpha diversity and changes in microbial community structure.

**Figure 8:**
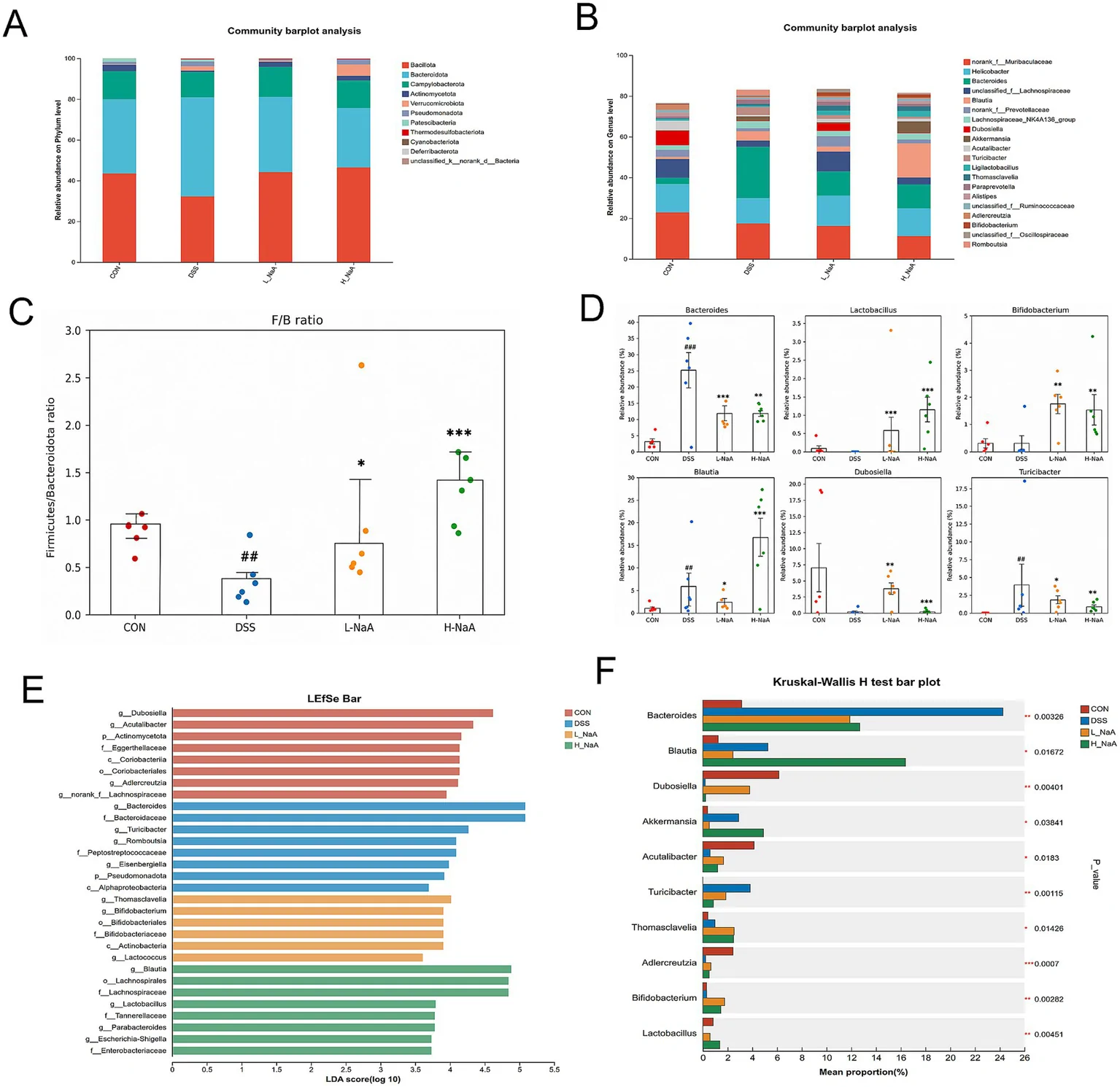
NaA remodels DSS-induced gut microbiota composition and modulates key differential taxa. **(A)** Bar plots showing the composition of gut microbiota at the phylum level in each group. **(B)** Bar plots showing the composition of gut microbiota at the genus level in each group. **(C)** Comparison of the Firmicutes/Bacteroidota (F/B) ratio among groups. **(D)** Relative abundances of representative differential genera, including *Bacteroides*, *Lactobacillus*, *Bifidobacterium*, *Blautia*, *Dubosiella*, and *Thomasclavelia*. **(E)** LEfSe showing differentially enriched taxa in each group. **(F)** Bar plots of representative differential genera identified by Kruskal–Wallis H test. Data are presented as mean ± SD (*n* = 6). ##*p* < 0.01, ###*p* < 0.001 compared to Control group; *p < 0.05, **p < 0.01, ***p < 0.001 compared to DSS group.

At the phylum level, community composition analysis revealed that DSS treatment significantly decreased the relative abundance of Firmicutes while increasing Bacteroidota compared with the control group. NaA intervention reversed these changes in a dose-dependent manner, with high-dose NaA showing the most pronounced effect (Figure 8A). At the genus level, DSS treatment caused visible shifts in the abundance patterns of several dominant genera, including *Bacteroides*, *Lactobacillus*, *Bifidobacterium*, *Blautia*, *Dubosiella*, and *Turicibacter*, whereas NaA intervention partially reshaped these genus-level alterations (Figure 8B). Further analysis demonstrated that the Firmicutes/Bacteroidota (F/B) ratio was significantly reduced in DSS-treated mice, whereas NaA significantly increased the ratio, with the high-dose group achieving the highest restoration (Figure 8C). The relative abundances of Firmicutes and Bacteroidota and the calculated F/B ratios for individual samples are provided in Supplementary Table S3. At the genus level, DSS markedly increased the abundance of *Bacteroides*, which was significantly reduced following NaA administration. Low-dose NaA notably enriched *Bifidobacterium*, whereas high-dose NaA substantially increased *Lactobacillus* and *Blautia* (Figure 8D). Other genera, including *Dubosiella* and *Thomasclavelia*, also displayed significant differences across groups, indicating that NaA influences multiple key taxa. LEfSe analysis identified representative dominant taxa for each group: *Bacteroides* and *Turicibacter* were enriched in the DSS group, Bifidobacterium was enriched in the low-dose NaA group, and *Blautia*, *Lactobacillus*, and several Lachnospiraceae-related genera were enriched in the high-dose NaA group (Figure 8E). Kruskal–Wallis tests further confirmed that the relative abundances of *Bacteroides*, *Blautia*, *Dubosiella*, *Akkermansia*, *Turicibacter*, *Thomasclavelia*, *Bifidobacterium*, and *Lactobacillus* differed significantly among the groups (Figure 8F).

Collectively, these findings indicate that NaA not only improves DSS-induced alterations in overall gut microbial community structure but also selectively remodels multiple key differential taxa, highlighting its potential to restore intestinal microbial homeostasis.

## 4 Discussion

UC is a chronic, relapsing inflammatory bowel disease. Although current therapies have expanded from conventional anti-inflammatory drugs to immunosuppressants and biologics, high recurrence rates and long-term safety limitations remain significant concerns. Therefore, identifying novel interventions that simultaneously target inflammation, restore epithelial barrier integrity, and maintain microbial homeostasis remains of substantial clinical relevance (Liang et al., 2024; Appiah et al., 2025). Acetate, the most abundant short-chain fatty acid in the colon, not only participates in energy metabolism but is also closely associated with epithelial barrier homeostasis and mucosal immune regulation. Consequently, NaA was selected as the intervention agent based on a solid theoretical rationale. *In vitro* experiments indicate that NaA exerts a clear protective effect on intestinal epithelial cells within a certain concentration range.

In the present study, NaA at 10–40 mM showed no significant cytotoxicity in NCM460 cells and effectively upregulated the expression of ZO-1 and Occludin. Under LPS-induced inflammatory conditions, NaA partially reversed the decline of these barrier proteins in both NCM460 and Caco-2 cells, indicating a consistent protective effect across different intestinal epithelial cell models. When the concentration increased to 80 mM, its protective effect diminished, suggesting that NaA acts in a concentration-dependent manner. Previous studies have also reported that in UC patient-derived organoid monolayers, 100 mM acetate increased transepithelial electrical resistance (TEER), reduced IL-8 and TNF-*α* expression, and upregulated HIF1α, MUC2, and MKI67 (Deleu et al., 2023); In contrast, butyrate did not consistently restore barrier function in primary epithelial monolayers under inflammatory conditions, and in some cases even exacerbated damage (Vancamelbeke et al., 2019). These findings suggest that, compared with butyrate, acetate may exhibit better tolerance and more stable protective potential in damaged epithelial environments, which is consistent with its predominance and relatively high physiological concentration in the colonic lumen (Fu et al., 2025).

The *in vivo* experimental results further support this conclusion at the systemic level. It should be noted that the effects of acetate in experimental colitis are highly dependent on both the administration route and concentration. Previous studies constructing colitis models with acetate often employed high-concentration local rectal administration, which essentially induces direct chemical injury and can cause acute mucosal damage within a short period (Subramanian et al., 2022); In contrast, the present study utilized oral gavage of 150 mM and 300 mM NaA as a pretreatment combined with DSS to induce colitis (Masui et al., 2013; Macia et al., 2015), a scenario that more closely mimics the modulatory effects of exogenous acetate in the gut following dilution, transport, and interaction with the host–microbiota ecosystem, rather than simple localized corrosive injury.

Therefore, acetate can induce mucosal damage under high local exposure, but at appropriate concentrations and administration routes, it can exert protective effects. Indeed, previous reports have shown that supplementation with 300 mM acetate can alleviate clinical scores and histological damage in DSS-induced colitis (Macia et al., 2015). In the present study, NaA mitigated body weight loss, colon shortening, DAI elevation, and splenic enlargement, further supporting its protective role under physiologically relevant conditions.

This study further demonstrates that the protective effects of NaA are closely associated with both inflammation suppression and barrier restoration. DSS treatment significantly elevated the levels of IL-6, TNF-*α*, and IL-1β, which were markedly reduced following NaA intervention. Concurrently, histological analysis and assessment of barrier-related proteins indicated that NaA alleviated tissue damage and restored the expression of ZO-1, Occludin, and MUC2. These two types of changes are not independent. TNF-*α*, IFN-*γ*, and IL-1β are among the inflammatory mediators most closely linked to increased intestinal epithelial permeability, capable of inducing tight junction remodeling and sustained barrier dysfunction (Kaminsky et al., 2021; Xiao et al., 2024); The inflammatory microenvironment can further impair barrier integrity by disrupting epithelial junction remodeling and mucosal repair processes (Luissint et al., 2016). Therefore, NaA not only suppresses local inflammatory responses but also mitigates secondary barrier damage mediated by inflammation. Moreover, the protective effects of NaA extend beyond the mechanical barrier to include preservation of the mucus barrier. MUC2 is the principal component of the colonic mucus layer, with goblet cells and their secreted mucin forming the first line of defense against direct bacterial contact with the epithelium. Reduction in MUC2 secretion or damage to the mucus layer facilitates bacterial adhesion to the epithelium and amplifies inflammatory responses (Yao et al., 2021; Kang et al., 2022). Grondin et al. (2020) reported that inflammatory cytokines such as IL-1β, TNF-α, and IFN-γ not only impair tight junction proteins but also interfere with mucin synthesis and goblet cell function, thereby compromising both mechanical and chemical barriers simultaneously. Mechanistically, the concurrent improvement in inflammation and barrier integrity by NaA may be related to acetate-specific receptor-dependent signaling and inflammasome modulation. Previous studies have shown that acetate can downregulate pro-inflammatory cytokines, including IL-8 and TNF-α, while promoting the expression of MUC2 and related repair molecules in inflammatory epithelial models (Deleu et al., 2023); SCFAs also participate in inflammatory regulation through GPR43 and HDAC/NF-κB signaling pathways (Du et al., 2024). Additionally, acetate can promote NLRP3 ubiquitination and autophagic degradation via a GPR43-mediated Ca^2+^-dependent mechanism, thereby inhibiting the release of IL-1β, IL-18, TNF-α, and IL-6 (Xu et al., 2019). Taken together, NaA mitigates DSS-induced colonic mucosal injury at multiple levels by suppressing inflammation amplification, reducing tight junction protein damage, and maintaining mucus layer integrity.

In this study, NaA intervention markedly attenuated the DSS- or LPS-induced increase in the BAX/BCL-2 ratio and activation of cleaved-caspase-3, suggesting that its protective effects are accompanied by the alleviation of excessive apoptosis in intestinal epithelial cells. This phenomenon is unlikely to be merely a secondary consequence of reduced inflammation. Previous studies have indicated that dysregulated epithelial apoptosis is a key pathological event in the onset and progression of UC. Under pathological conditions, the normally ordered process of proliferation, differentiation, and shedding along the crypt–villus axis is disrupted. Premature death of mature epithelial cells can directly compromise epithelial continuity, increase barrier permeability, and facilitate bacterial and antigen translocation, thereby amplifying local inflammatory responses (Wu et al., 2025). Based on this understanding, the restoration of ZO-1, Occludin, and MUC2 by NaA in the present study is likely not solely a direct effect. Rather, it may reflect the structural support provided by NaA through the reduction of pathological epithelial cell loss, which underlies the maintenance of both the mechanical and mucus barriers.

Mechanistically, the observed changes in BAX/BCL-2 ratio and cleaved-caspase-3 suggest that NaA may act on the mitochondria-dependent apoptotic pathway. Previous studies have indicated that imbalance of Bcl-2 family proteins can increase mitochondrial outer membrane permeability, promote the release of cytochrome c and initiate the caspase-9/caspase-3 cascade, thereby driving apoptosis. Concurrently, sustained endoplasmic reticulum (ER) stress can upregulate BAX and downregulate BCL-2 via the PERK/ATF4/CHOP and IRE1*α*/ASK1/JNK signaling axes, creating significant crosstalk with mitochondrial apoptosis pathways (Pihán et al., 2017; Chen et al., 2023). Therefore, the reduction in the BAX/BCL-2 ratio and cleaved-caspase-3 levels observed in this study may not merely indicate inhibition of terminal apoptotic execution; rather, it suggests that NaA may ameliorate upstream pro-apoptotic stress conditions. Supporting this notion, Shen et al. (2016) demonstrated in a DSS-induced colitis model that acetate-related interventions simultaneously suppressed the expression of Bax, caspase-3/8/9, and multiple ER stress-related molecules, indicating that the protective effects of acetate in experimental colitis are closely linked to attenuation of ER stress-mediated apoptosis.

It is noteworthy that the formation of a pro-apoptotic environment is not limited to ER stress alone; oxidative stress and inflammatory signaling also contribute. In UC, excessive accumulation of ROS can disrupt mitochondrial homeostasis, aggravate epithelial energy metabolism disorders, and increase the tendency toward apoptosis. Meanwhile, sustained elevation of inflammatory cytokines such as TNF-α, IL-1β, and IL-6 may further amplify epithelial injury (Wan et al., 2022). Related studies have shown that, in UC models, improvement of oxidative stress and downregulation of NF-κB-related inflammatory signaling are accompanied by decreased levels of mitochondrial apoptosis-related molecules, including Cyt-c, caspase-9, and caspase-3, suggesting a close relationship among oxidative stress, inflammatory responses, and mitochondrial apoptosis (Shen et al., 2019). In addition, the association between apoptosis alleviation and barrier repair has also been indirectly supported by other colitis studies, in which reduced apoptosis is often accompanied by decreased intestinal permeability, restoration of junctional proteins, and increased goblet cell numbers (Pochard et al., 2021). Taken together with the present findings, it can be inferred that NaA may inhibit excessive intestinal epithelial apoptosis by reducing inflammation and upstream stress responses, thereby providing a structural basis for the restoration of the mechanical and mucus barriers.

Moreover, we noted that excessive apoptosis of epithelial cells not only directly compromises barrier integrity but can also alter the luminal microenvironment, thereby influencing the composition of the gut microbiota. Microbial dysbiosis is recognized as a critical factor in the onset and progression of UC, and such ecological imbalance can further impact mucosal inflammation, epithelial barrier integrity, and host immune homeostasis (Ni et al., 2017). In the present study, DSS treatment caused a marked shift in overall microbial community structure, which was partially corrected by NaA intervention. These findings suggest that the protective effects of NaA in experimental colitis are, at least in part, mediated by remodeling of the gut microbiota, rather than being solely dependent on direct suppression of host inflammatory responses.

At the phylum level, we observed that the Firmicutes/Bacteroidota (F/B) ratio was markedly reduced in the DSS group, whereas NaA, particularly at high doses, promoted its recovery. Previous studies have suggested that Firmicutes contribute to gut homeostasis through the production of microbial metabolites, thereby enhancing gastrointestinal barrier integrity and mucosal immune function (Qiu et al., 2022). In contrast, relative expansion of Bacteroidota under inflammatory conditions is often associated with microbial dysbiosis (Ni et al., 2017; Glassner et al., 2020). Accordingly, in many prior studies, a decrease in the F/B ratio has been considered a general indicator of colitis-associated microbial imbalance, and restoration of the F/B ratio following treatment is regarded as evidence of microbial community reconstruction (Zhou et al., 2025). Based on these considerations, the improvement of the F/B ratio by NaA in the present study suggests that it may partially reverse DSS-induced alterations in overall gut microbial community structure.

Further analysis at the genus level revealed that DSS treatment markedly increased the relative abundances of *Bacteroides* and *Turicibacter*, whereas NaA intervention significantly reduced their levels. Although Bacteroidota are closely involved in acetate and propionate metabolism (Liu et al., 2023), certain *Bacteroides* strains can act as pathobionts under inflammatory conditions (Zafar and Saier, 2021), Wang et al. (2019) also reported that excessive *Bacteroides* in the gut is considered detrimental to the intestinal immune system, and its abnormal expansion likely reflects enhanced inflammation rather than protective effects. *Turicibacter* is generally regarded as a pro-inflammatory genus, showing high abundance in UC patients. In animal models of UC, *Turicibacter* overgrowth has been associated with exacerbated intestinal injury and severe complications (Dong et al., 2022; Jia et al., 2025), and it may promote colorectal carcinogenesis through mechanisms such as chronic inflammation, DNA damage, and the production of bioactive carcinogenic metabolites (Chung et al., 2021), In the present study, NaA intervention significantly suppressed the abnormal expansion of *Turicibacter*, concomitant with reduced inflammation and restoration of barrier integrity. By contrast, the recovery of *Bifidobacterium*, *Lactobacillus*, and *Blautia* following NaA intervention is more indicative of protective effects. *Bifidobacterium* is a typical acetate-associated genus that participates in acetate production and can support the growth of other SCFA-producing bacteria via cross-feeding interactions (Xiao et al., 2024); Notably, Fukuda et al. (2011) demonstrated that acetate derived from *Bifidobacterium* reduces epithelial cell death, maintains transepithelial barrier integrity, and limits toxin translocation. In this study, enrichment of Bifidobacterium in the low-dose NaA group was associated with changes in bacterial taxa previously linked to acetate metabolism. *Lactobacillus*, a common probiotic, typically decreases under UC or DSS-induced colitis, whereas its restoration after intervention is often associated with reduced inflammation, improved microbial homeostasis, and enhanced barrier function. Previous studies with Huangqin polysaccharides reported a significant decrease of *Lactobacillus* after DSS treatment, which was restored following therapy (Cui et al., 2021); similar observations were made in alginate oligosaccharide interventions, linking Lactobacillus recovery to correction of microbial dysbiosis and SCFA restoration (Wu et al., 2023). Research on vinegar or acetate supplementation has also reported alterations in Lactobacillus and Bifidobacterium abundance, which have been associated with microbial homeostasis and SCFA-related functions (Shen et al., 2016). In the present study, the increased *Lactobacillus* in the high-dose NaA group likely reflects the establishment of a colonic environment more conducive to SCFA-related metabolism and mucosal repair.

**Figure 9:**
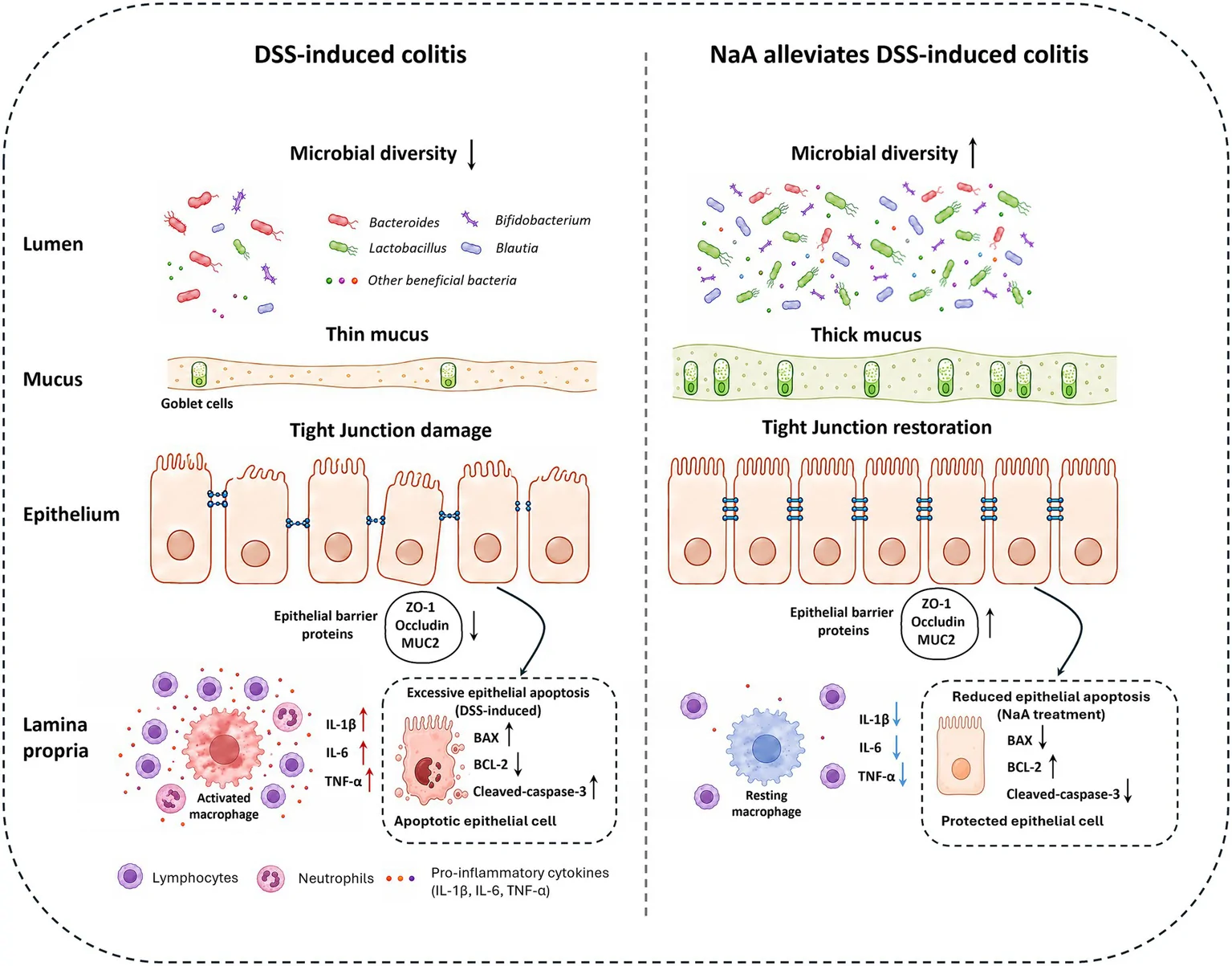
Schematic diagram of the protective mechanisms of NaA in DSS-induced ulcerative colitis.

*Blautia*, a genus within the Lachnospiraceae family, is closely linked to anaerobic fermentation and SCFA metabolism (Mukhopadhya and Louis, 2025), and exhibits multiple potential health-promoting effects. For example, specific species such as *Blautia wexlerae* can produce metabolites including acetate, lactate, and other small molecules, which improve obesity and diabetes phenotypes induced by high-fat diets while reducing inflammation (Hosomi et al., 2022); *Blautia coccoides* has been shown to promote colonic mucus layer growth through SCFA pathways, thereby maintaining barrier function (Holmberg et al., 2024). Moreover, intervention studies in DSS-induced colitis models have demonstrated that *Blautia* exhibits anti-inflammatory and antioxidative properties, mitigating inflammation and oxidative damage (Mao et al., 2024). Although NaA differentially modulates specific genera depending on the dose, the overall microbial changes are associated with taxa previously linked to SCFA metabolism and intestinal homeostasis. The proposed protective mechanisms of NaA in DSS-induced ulcerative colitis are summarized schematically in Figure 9. Despite these findings, several limitations should be acknowledged. First, although this study demonstrated the protective effects of NaA in a DSS-induced mouse colitis model, this acute experimental model cannot fully recapitulate the chronic and heterogeneous characteristics of human UC. Further validation using chronic colitis models and clinical samples is warranted. Second, although 16S rRNA sequencing showed that NaA reshaped gut microbiota composition, these results indicate an association rather than a causal relationship between microbial changes and colitis improvement. Future studies using fecal microbiota transplantation, antibiotic-treated mice, SCFA measurements, and multi-omics analyses are needed to clarify whether specific microbial alterations directly contribute to the protective effects of NaA. Finally, although NaA was well tolerated during the experimental period, comprehensive safety evaluations were not performed. Moreover, differences in intestinal metabolic environments between experimental models and humans may influence the translational relevance of NaA supplementation. Further studies are required to define its long-term safety and clinical applicability.

## 5 Conclusion

In conclusion, this study demonstrates that NaA ameliorates colitis-associated damage by attenuating inflammatory responses, inhibiting excessive apoptosis of intestinal epithelial cells, restoring both mechanical and mucus barriers of the colonic mucosa, and partially correcting gut microbiota dysbiosis. These findings indicate that NaA exerts a protective effect in UC and provide experimental evidence supporting the potential application of acetate in the prevention and treatment of this disease.

## Data Availability

The raw 16S rRNA gene sequence reads generated in this study have been deposited in the NCBI Sequence Read Archive (SRA) under BioProject accession number PRJNA1468164.
