# Supplementary material for: Sodium acetate attenuates ulcerative colitis by reducing intestinal epithelial apoptosis and remodeling gut microbiota
Source: Front Microbiol. 2026 Aug 20;17:1888741. doi: 10.3389/fmicb.2026.1888741 (PMC13538854; doi:10.3389/fmicb.2026.1888741)
Supplement: Supplementary file 1 [file Supplementary_file_1.DOCX]

Supplementary Material

Supplementary tables

Table S1. Reagents, kits, and antibodies used in this study

| Reagent/Antibody | Supplier | Catalog No. | Working concentration |
| --- | --- | --- | --- |
| NCM460 cells | Procell | CL-0749 |  |
| Caco-2 | Procell | CL-0050 |  |
| NaA | Yuanye | V33678 |  |
| LPS | Beyotime | S1732 |  |
| DMEM | Procell | PM150210 |  |
| FBS | Procell | 164210 |  |
| Penicillin-streptomycin solution | Procell | PB180120 |  |
| PBS | Procell | PB180327 |  |
| Trypsin-EDTA | Procell | PB180219 |  |
| CCK-8 kit | Solarbio | CA1210 |  |
| TRIzol reagent | Vazyme | R411-01 |  |
| RNA extraction reagent | Accurate Biology | AG21101 |  |
| Reverse transcription reagent/kit | Accurate Biology | AG11705 |  |
| RT-qPCR reagent/SYBR Green premix | Accurate Biology | AG11701 |  |
| BCA Protein Assay Kit | Elabscience | E-BC-K318-M |  |
| ECL chemiluminescent detection kit | Affinity Biosciences | KF8001 |  |
| DSS | Meilun Bio | MB5535 |  |
| Mouse IL-6 ELISA kit | Ruixin Biotech | JRXW201886 |  |
| Mouse TNF-α ELISA kit | Ruixin Biotech | JRXW208526 |  |
| Mouse IL-1β ELISA kit | Ruixin Biotech | JRXW201746 |  |
| primary ZO-1 antibody | Abcam | ab96587 | WB 1：2000 IHC 1：500 |
| Primary Occludin antibody | Proteintech | 27260-1-AP | WB 1：5000 IHC 1：1000 |
| MUC2 | Cell Signaling Technology | 16564 | IHC 1：1000 |
| BAX | Cell Signaling Technology | 2772 | 1：1000 |
| BCL2 | Cell Signaling Technology | 3498 | 1：1000 |
| Cleaved-caspase3 | Cell Signaling Technology | 9661 | 1:1000 |
| GAPDH | Huabio | HA724237H | 1:2000 |
| HRP-conjugated secondary antibodies for IHC | BOSTER | SV0002 | 1:500 |
| HRP-conjugated secondary antibodies for WB | Absin | abs20213 | 1:20000 |
| Tris-EDTA buffer | Solarbio | C1038 |  |
| goat serum | Solarbio | SL038 |  |
| DAB Substrate Kit | Solarbio | DA1010 |  |
| hematoxylin | Solarbio | G1080 |  |
| neutral resin | Solarbio | G8590 |  |
| RIPA buffer | Solarbio | R0010 |  |
| PMSF | Solarbio | IP0280 |  |
| Three-color Prestained Protein Marker | Epizyme | WJ103 |  |
| loading buffer | Solarbio | P1040 |  |
| Rapid SDS-PAGE Gel Preparation Kit | Epizyme | PG222 |  |
| Ice-Free Fast Transfer Buffer | Epizyme | PS201 |  |
| Tris-Glycine SDS-PAGE Running Buffer | Servicebio | G2018 |  |
| 4% paraformaldehyde | RWD | R510-22-10 |  |

Table S2. Histological damage scoring criteria for H&E-stained colon sections.

| score | Goblet cell loss | Mucosal structure destruction | Muscle thickening | Inflammatory cell infiltration | Crypt abscess formation |
| --- | --- | --- | --- | --- | --- |
| 0 | None | Normal mucosal structure | Normal | None or normal | None |
| 1 | Mild | Mild mucosal damage | Mild | Mild inflammatory cell infiltration | Mild |
| 2 | Moderate | Moderate mucosal destruction | Moderate | Moderate inflammatory cell infiltration | Moderate |
| 3 | Severe | Severe mucosal destruction | Severe | Severe inflammatory cell infiltration | Severe |

Table S3. Relative abundance of Firmicutes and Bacteroidota and calculated F/B ratio in individual samples.

| No. | Sample ID | Group | Firmicutes/Bacillota relative abundance (%) | Bacteroidota relative abundance (%) | F/B ratio |
| --- | --- | --- | --- | --- | --- |
| 1 | m1 | CON | 26.2990 | 26.9737 | 0.9750 |
| 2 | m2 | CON | 26.9476 | 28.3200 | 0.9515 |
| 3 | m3 | CON | 27.0650 | 44.3673 | 0.6100 |
| 4 | m4 | CON | 25.7025 | 32.7075 | 0.7858 |
| 5 | m5 | CON | 31.6416 | 29.4771 | 1.0734 |
| 6 | m6 | CON | 39.5560 | 41.8443 | 0.9453 |
| 7 | m7 | DSS | 16.8264 | 65.0694 | 0.2586 |
| 8 | m8 | DSS | 18.9191 | 62.1748 | 0.3043 |
| 9 | m9 | DSS | 13.8992 | 66.8362 | 0.2080 |
| 10 | m10 | DSS | 25.8198 | 32.0816 | 0.8048 |
| 11 | m11 | DSS | 31.4069 | 38.2554 | 0.8210 |
| 12 | m12 | DSS | 24.6496 | 54.5570 | 0.4518 |
| 13 | m13 | L-NaA | 27.1660 | 41.5184 | 0.6543 |
| 14 | m14 | L-NaA | 56.1771 | 21.3117 | 2.6360 |
| 15 | m15 | L-NaA | 25.2885 | 43.8686 | 0.5765 |
| 16 | m16 | L-NaA | 26.2273 | 43.9370 | 0.5969 |
| 17 | m17 | L-NaA | 37.3492 | 42.8907 | 0.8708 |
| 18 | m18 | L-NaA | 22.0288 | 45.8537 | 0.4804 |
| 19 | m19 | H-NaA | 28.7144 | 30.1812 | 0.9514 |
| 20 | m20 | H-NaA | 39.0052 | 22.1951 | 1.7574 |
| 21 | m21 | H-NaA | 34.0146 | 39.0638 | 0.8707 |
| 22 | m22 | H-NaA | 41.8737 | 30.4355 | 1.3758 |
| 23 | m23 | H-NaA | 44.5759 | 26.0512 | 1.7111 |
| 24 | m24 | H-NaA | 39.2920 | 26.0904 | 1.5060 |

2.2 Figures

LEfSe analysis

PICRUSt2 functional prediction

Occludin expression in NCM460 cells induced by different concentrations of NaA

ZO-1 expression and GAPDH in NCM460 cells induced by different concentrations of NaA

ZO-1 expression and GAPDH in NCM460 cells induced by different concentrations of LPS

Occludin expression and GAPDH in NCM460 cells induced by different concentrations of LPS

NaA alleviates the LPS-induced reduction of ZO-1 expression

NaA alleviates the LPS-induced reduction of Occludin expression

Apoptotic molecule BCL2 in NCM460

BAX in NCM460

Cleaved-caspase3 in NCM460

ZO-1 and Occludin expression in Caco-2

cleaved-caspase3,BCL-2 and BAX expression in Caco-2

ZO-1 expression in animal tissues

Occludin expression in animal tissues
